# Primary Nursing in Intensive Care Units

**DOI:** 10.1111/nicc.70325

**Published:** 2026-01-20

**Authors:** Lars Krüger, Thomas Mannebach, Francesco Squiccimarro, Laura‐Carina Kurz, Christian Höke, Almut Pörner, Benjamin Sarx, Christian Siegling, Esther Mertins, Tobias Becker, René Schramm, Jan Gummert, Volker Rudolph, Gero Langer, Franziska Wefer

**Affiliations:** ^1^ Project and Knowledge Management/Care Development Intensive Care, Care Directorate, Heart and Diabetes Center NRW, Ruhr University Bochum Bad Oeynhausen Germany; ^2^ Institute of Health, Midwifery and Nursing Sciences, Medical Faculty, Martin Luther University Halle‐Wittenberg Halle (Saale) Germany; ^3^ Care Development, Care Directorate, Heart and Diabetes Center NRW, Ruhr University Bochum Bad Oeynhausen Germany; ^4^ Surgical Intensive Care Unit, Heart and Diabetes Center NRW, Ruhr University Bochum Bad Oeynhausen Germany; ^5^ Cardiology/Angiology Intensive Care Unit, Heart and Diabetes Center NRW, Ruhr University Bochum Bad Oeynhausen Germany; ^6^ Care Directorate, Heart and Diabetes Center NRW Ruhr University Bochum Bad Oeynhausen Germany; ^7^ Clinic for Thoracic and Cardiovascular Surgery, Heart and Diabetes Center NRW, Ruhr University Bochum Bad Oeynhausen Germany; ^8^ Clinic for General and Interventional Cardiology/Angiology, Heart and Diabetes Center NRW, Ruhr University Bochum Bad Oeynhausen Germany

**Keywords:** change management, nursing, nursing process, patient care planning, patient‐centred care

## Abstract

**Background:**

As a patient‐centred model of nursing care, primary nursing (PN) ensures continuity of care while promoting systematic involvement of patients and family members in the therapeutic process. To date, comprehensive published research projects that address the implementation of PN on intensive care units (ICUs) are rare.

**Aims:**

Primary aim was to evaluate the overall process of development and implementation of PN in two German ICUs of a university hospital. Secondary aims were to identify changes on ICU, as well as nursing performance indicators.

**Study Design:**

Quantitative design on a surgical (ICU 1) and medical (ICU 2) ICU. We used the validated German *Instrument zur Erfassung von Pflegesystemen* (*IzEP(c)*) with separate questionnaires for patients, relatives and medical staff at three data collection points as an as‐is analysis before (t_0_), after six (t_1_) and 12 months (t_2_) of implementation of PN in practice. IzEP(c) enables a percentage calculation of the practiced nursing organisation model (overall ICU profile; PN at > 75%), information of the ICU profile (e.g., communication) and nursing performance indicators (e.g., patient participation). For descriptive statistics, a programmed Microsoft Excel spreadsheet with built‐in percentage calculations, developed by the IzEP(c) development team, was used.

**Results:**

Data collection took place between September 2023 and March 2025, and 264 questionnaires were analysed. The overall profile on ICU 1 started with individual nursing (44.5%, t_0_) up to PN in t_2_ (83.0%). ICU 2 reached individual nursing between t_0_‐t_2_ with characteristics toward PN (t_0_: 51.0%; t_2_: 69.0%). Between t_0_‐t_2_, ICU profile showed good development in communication in ICU 1 (36.0%; 77.0%) and necessary change in ICU 2 (38.5%; 46.5%). Nursing performance indicators reached good development in both ICUs with development potential in, for example, patient participation in ICU 2 (54.0%; 49.5%).

**Conclusions:**

PN was practiced in all included patients in both ICUs, but implementation was not fully achieved in ICU 2. Nevertheless, PN was practiced in included patients on ICU 2. Another evaluation on ICU 2 should be planned.

**Relevance to Clinical Practice:**

PN on ICU is feasible and needs continuous support from nursing managers for successful implementation. An accompanying evaluation is mandatory for this purpose.

**Trial Registration:**

This study is registered at the German Clinical Trials Register as DRKS00030966.

## Impact Statements

- What is known about this topic?
  ○ Primary nursing (PN) is a nursing organisation model that is practiced internationally in intensive care units (ICUs).
  ○ There are first‐quality development projects that address the implementation of PN using the validated tool *Instrument zur Erfassung von Pflegesystemen* (*IzEP(c)*) in ICUs in Germany, Austria and Switzerland.
- What this paper adds?
  ○ This study focuses on the implementation process of PN in a surgical (ICU 1) and medical (ICU 2) ICU with altogether 48 beds in a German university hospital using IzEP(c).
  ○ While PN was practiced in all included patients, results showed a well‐implemented PN in ICU 1 and further necessary development in ICU 2.
  ○ Nursing performance indicators of both ICUs reached well results with development potential in, for example, patient participation in ICU 2.

## 1 Introduction

Different nursing organisation models are described for intensive care units (ICUs). They can be divided into task‐oriented and person‐centred approaches [1]. However, there are different variations, particularly in the context of person‐centred care. Primary nursing (PN) is described as a person‐centred care model [1] and thus also includes close involvement of the family [2, 3, 4].

## 2 Background

Functional nursing was primarily practiced in the early 1930s and focuses on task orientation. Nurses are responsible for special tasks like blood pressure measurement in every patient in their unit, while the head nurse is responsible for the overall nursing care process [1]. In the following years, nursing organisation evolved toward increasingly patient‐centred care. In individual nursing, a nurse provides holistic care for assigned patients during one shift. The nursing leader also assumes responsibility for the nursing care process here [1].

In the late 1960s, Marie Manthey and her team developed PN in the USA [5]. PN is based on four core elements, which have also been considered separately in nursing management since 2015: responsibility for relationships and decision‐making, work allocation and assignment of patients, communication among employees, management and leadership philosophy [6]. PN ideally starts upon admission to hospital [1]. The primary nurse's responsibility for the nursing care process also results in a bond and correspondingly close communication with relatives [7, 8]. This is in line with one of the recommendations of the current guideline of family‐centred care for adult ICUs [9] and also helpful for visits by children in ICU [10, 11].

Subsequently, some quality development projects were described and first and initial findings from implementation efforts published. To date, these also include ICUs in different countries [7, 12, 13, 14]. Goode and Rowe [7] used focus group interviews (FG) to evaluate implementation of PN in a Northern Irish ICU. Positive impacts on families have been identified, attributable to the primary nurse's sustained responsibility and continuity in delivering care [7]. Fröhlich et al. [13] evaluated the implementation of PN in Switzerland using the validated German *Instrument zur Erfassung von Pflegesystemen* (*IzEP(c)*) on three ICUs which also included questionnaires of relatives. As a result, continuity in nursing care improved, but it was not always easy for relatives to identify the primary nurse. The authors discussed a possible influence from frequent shift changes of nurses in this context [13]. Chen et al. [14] described a quality development project in Chinese hospitals, which also included ICUs. Functional nursing was practiced before and PN had previously been implemented. As one result, patients rated better communication between nurses and themselves [14]. Krüger et al. [12] implemented PN in a German ICU. In FG, nurses reported that PN helps to better integrate relatives into nursing care [8]. In a further randomised feasibility study, relatives ranked a better influence on patient care and nurse contact in comparison to individual nursing [15].

### 2.1 Rationale

To date, there is only one description of the development and implementation process of PN in an ICU in Germany. As part of a nursing quality development project, PN as a person‐centred nursing organisation model was to be developed and implemented in two ICUs at a German university hospital. The process was evaluated using a mixed‐methods design.

### 2.2 Aims

Primary aim of this study was to evaluate the process of development and implementing PN in ICU 1 and ICU 2. Secondary aims were to identify changes in the ICU profiles as well as nursing performance indicators.

## 3 Methods

**FIGURE 1:**
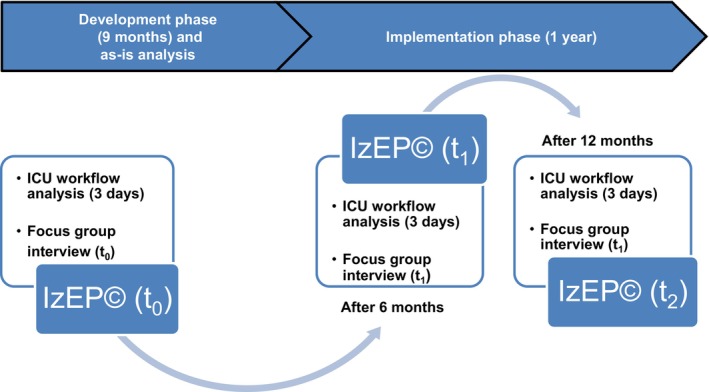
Overview of the evaluation of implementation primary nursing. ICU, Intensive care unit; IZEP(c), Instrument zur Erfassung von Pflegesystemen.

For overall evaluation we used a mixed‐methods design as an as‐is analysis (t_0_), after six (t_1_) and 12 months (t_2_) (Figure 1). Qualitatively, an FG with six nurses and an ICU workflow analysis on 3 days in various day shifts was used at each data collection timepoint. This paper focuses on the quantitative evaluation.

The study report follows the Standards for Quality Improvement Reporting Excellence Guideline (Squire 2.0) [16] (Table S1). In developing PN roles, the process was aligned with the Medical Research Council's Complex Intervention Framework (MRC Framework), focusing on the first two stages: developing the intervention and assessing feasibility [17]. For implementation and change management we followed the recommendations of the updated Consolidated Framework for Implementation Research (CFIR) with the five domains: innovation, outer setting, inner setting, individuals and implementation process [18].

### 3.1 Setting

This study was conducted at the Heart and Diabetes Center NRW, a German university hospital of the Ruhr University Bochum with six ICUs. Two ICUs with medical specialisations, Thoracic and Cardiovascular Surgery (25 beds, ICU 1) and General and Interventional Cardiology/Angiology (23 beds, ICU 2), were included. Before the start of this study as a quality improvement project, individual nursing was practiced as a nursing organisation model. PN had already been successfully implemented in a first ICU in 2022. Results were published elsewhere [8, 12].

In both ICUs, patients are treated after all types of thoracic and cardiovascular surgery, including ventricular assist devices and heart transplantation, as well as conventional cardiological and cardiac electrophysiology. A nurse‐to‐patient ratio of 1:2 is practiced. Nurses have at least 3 years of basic nursing training (registered nurses; RN). Some nurses also have a German state‐certified further training course in intensive and anaesthesia care nursing (ICU training), a Bachelors or Masters degree in nursing or further training. The nursing management team of both ICUs practices a shared governance model with three areas of focus: nursing management, nursing training and nursing science [19].

### 3.2 Recruitment and Data Collection

In accordance with the specifications of IzEP(c) [20] we planned to invite 32 participants and additionally include 6 patient documentations and 6 duty rosters of nurses in each timepoint and ICU. The time period for data collection was planned over 18 months. All participants were personally informed by the study leaders or specially trained nurses. The informed written consent of patients was first obtained before inviting their relatives to take part in the study. After written informed consent, all participants were included. Subsequently, specially trained nurses conducted face‐to‐face interviews with the participants using the IzEP(c) questionnaires. Since the questionnaires vary in length in each target group, it took between 5 (e.g., physicians) and 20 (nurses) minutes to complete them [20].

### 3.3 Intervention Primary Nursing

The tasks of primary nurses and nurses without nursing process responsibility (associated nurses) were developed in separate working groups in both ICUs over a period of 9 months (Figure 1). During this time, working groups met every second week for 2 h. Continuous information for all nurses and the interdisciplinary team was presented using weekly team meetings and a 24‐h continuously automatically presented PowerPoint presentation on TV in the break room. Afterwards, task profiles were approved by the nursing team by separate consensus conferences in both ICUs (Table S2). This was based on the four core elements of PN. Nursing management and staff representatives were also involved in this process. PN in ICU 1 and ICU 2 is initiated on the third day of the patient's ICU stay and involves the use of a self‐developed nursing assessment tool [12, 21], the structured management of the nursing process with a written nursing care plan and the delivery of nursing care for up to two patients [12]. PN continues until transfer to the general ward, another hospital or the rehabilitation centre.

The foundation for further conditions of PN was agreed in 2022 [12]. These include an additional 45 min of working time twice a week for the preparation and evaluation of nursing planning and conversations with relatives, as well as a financial supplement for reduced night shifts (maximum of four per month). Primary nurses have to be qualified RNs with a minimum of 3 years of professional experience in intensive care. In addition, they should have completed advanced training in areas relevant to primary nursing, such as patient care planning, communication and the involvement of patients and relatives in the nursing care process. Ideally, they should also hold ICU training or an academic degree at the bachelor's level [12]. The job scope should ideally be full‐time (38.5 h), but at least 20–25 h per week. In case of part‐time positions, a primary nursing tandem is planned and implemented to ensure continuity of nursing care. In addition, primary nurses represent a close link to the interdisciplinary team, but also to the relatives. Patients and relatives were informed about PN personally by primary nurses with an additional flyer. Associated nurses represent the primary nurses in their absence and follow their nursing plan. PN has been practiced since February 2024 in both ICUs.

### 3.4 Evaluation and Sample

We used version 3.0 of the IzEP(c) which consists of 294 items in nine different questionnaires with single‐choice questions [20, 22]. Reliability [23] and validity of IzEP(c) were validated in German language in various settings [20] and it was also used on ICU [12, 13]. Content Validity Index (CVI) Atlas of Variant Effects Alliance was 0.95 and Universal Agreement 0.85 [22]. The IzEP(c) manual describes in detail the number of questionnaires required for each ICU. This is based on the number of staff and beds [20]. In each ICU, 25% of all nurses, 3 therapeutic professions, three external professional groups, nursing management of the ICU and care directorate, 25% of patients (at least 6), 50% of the patients' relatives, patient documentation and duty roster of nurses from all included patients must be considered [22, 23]. An example of a question for relatives was: ‘Which of the following statements best describes the situation of your relative?’ Relatives could then choose five answer options between ‘I don't know, I can't judge’ to ‘… that a single nurse is responsible for him for the entire stay’.

The overall IzEP(c) ICU profile provides an overview of the nursing organisation practiced in an ICU in a range from 0%–100%. Up to 10% there is no clear rule in nursing organisation, functional nursing is found in the range of 10%–40%, individual nursing in the range of 41%–75% and PN in the range of > 75%–100%. Furthermore, IzEP(c) enables a specific evaluation of dimensions for a ICU profile with responsibility and continuity, nursing care process, communication and understanding of roles [22]. Moreover, nursing performance indicators are available, such as formal completeness and documentation of the nursing process, formal structuring of communication, perception of the nursing care process by patients, patient participation and relationship with the primary nurse. The indicators are shown as percentages and indicate the degree of implementation in practice [20].

In context of ICU profiles which are divided into the four dimensions responsibility and continuity, nursing care process, communication and understanding of roles, IzEP(c) distinguishes between Feature A and Feature B. Feature A is to be interpreted as a conceptual specification determined by nursing management (target value), indicating which requirements are intended to be achieved in the respective ICU. Feature B reflects the current achieved situation in the practice of a ICU perceived by nurses, other health professionals, patients and relatives (actual value) [20]. If target values substantially exceed the actual values, management priority should be given to process development and team support. Conversely, if the actual values surpass the target values, a critical review of the conceptual requirements with adjustments is recommended [20].

### 3.5 Data Analysis

A programmed Microsoft Excel 2021 spreadsheet is available for evaluating the results of IzEP(c) questionnaires [24]. Data were transferred by two trained nurses in each ICU. For quality assurance purposes, an additional 10% sample was taken by a third nurse. Since no deviations were found, the data were then evaluated and presented with methods of descriptive statistics as percentages and, if possible, with numbers. IzEP(c) does not provide further data analyses and is limited to calculating percentages. The individual items and answers of participants in each questionnaire, for example, nurses, patients, relatives and physicians, are weighted differently in IzEP(c) profiles and a separate statistical evaluation would not take into account all of the calculated weightings. In the nurses' questionnaire alone, several items relate to the nursing organisation model applied in practice. For this reason, no further analyses could be carried out, as these would not have been appropriate in the context of the overall ICU profile.

### 3.6 Ethical Considerations

Written informed consent was given by all study participants. The local ethics committee of the medical faculty of the Ruhr University Bochum, Germany, based in East Westphalia, approved the study on November 18th 2022 (No 2022‐987). The study was subsequently registered in the German register of clinical trials (DRKS‐ID: DRKS00030966). All described investigations involving humans were carried out in accordance with national law and the Declaration of Helsinki in the current, revised version.

## 4 Results

Data were collected in September 2023 (t_0_), August 2024 (t_1_) and March 2025 (t_2_). In total, data from 264 IzEP(c) questionnaires with 192 included participants were incorporated into the data analysis. The top three of the participants in both ICUs were nurses (*n* = 90), patients (*n* = 36) and relatives (*n* = 18) (Table S3).

The overall ICU profile in ICU 1 started with 44.5% (t_0_) up to 73.0% in t_1_ and 83.0% in t_2_. ICU 2 reached 51.0% in t_0_, followed by 71.5% (t_1_) and 69.0% (t_2_) (Figure S4). Relatives gave heterogeneous answers in t_0_‐t_1_, while in t_2_ they stated that, for example, the same nurse was responsible across multiple shifts in both ICUs (Table S5). Nurses answered similarly to the same question in context of the practiced nursing organisation (Table S6). During the study period 80 (ICU 1) and 69 (ICU 2) patients were included in PN (Table S7).

**FIGURE 2:**
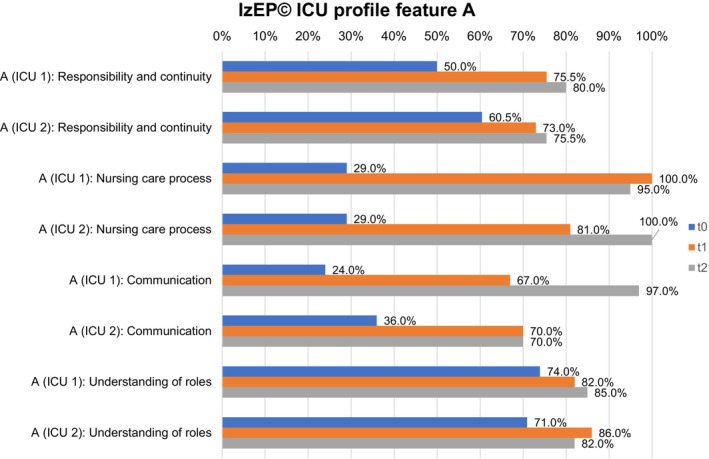
IzEP(c) ICU profile feature A for ICU 1 and ICU 2. ICU, Intensive care unit; IZEP(c), Instrument zur Erfassung von Pflegesystemen.

**FIGURE 3:**
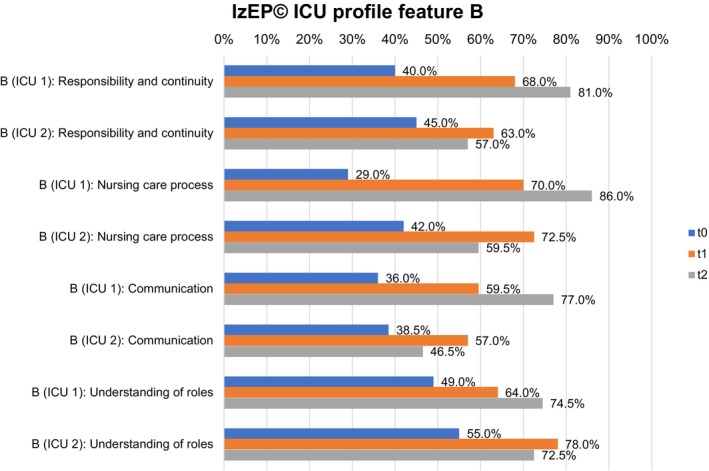
IzEP(c) ICU profile feature B for ICU 1 and ICU 2. ICU, Intensive care unit; IZEP(c), Instrument zur Erfassung von Pflegesystemen.

Feature A (Figure 2) and feature B (Figure 3) represent the ICU profiles of both ICUs in detail.

### 4.1 ICU Profiles

In both ICUs, feature A of dimension responsibility and continuity continued to develop at all three timepoints up to 80.0% (ICU 1) and 75.5% (ICU 2). Feature B reached 81.0% in ICU 1 and decreased from 63.0% (t_1_) to 57.0% (t_2_) in ICU 2. In particular, the change was evident in ICU 2, where the primary nurse was often not remembered by patients. Nursing records showed a heterogeneous presence of primary nurses, which increased as the study progressed.

The nursing care process in ICU 1 increased in both ICUs in feature A in t_1_. In t_2_ it decreased in ICU 1 (100% to 95.0%) and increased in ICU 2 to 100%. Feature B showed less pronounced manifestations in t_2_, but continuously increased to 86.0% in ICU 1 and decreased from 72.5% (t_1_) to 59.5% in ICU 2. The duty roster of ICU 1 showed a good presence of primary nurses in ICU 1. In ICU 2, time periods with fewer primary nurses were revealed, for example, because of illness or further training.

Communication in feature A increased in ICU 1 up to t_2_ (97.0%), while there was no change in ICU 2 between t_1_ and t_2_ (70.0%). Feature B showed an increase up to 77.0% (t_2_) in ICU 1, but a decrease between t_1_ (57.0%) and t_2_ (46.5%) in ICU 2. A detailed examination of the questionnaires showed generally positive feedback in both ICUs. In ICU 2, communication structures were rated less favourably in t_2_ compared to t_1_.

In both ICUs, understanding of roles increased in feature A between t_0_ and t_1_. While ICU 1 also increased up to 85.0% in t_2_, in ICU 2, it decreased from 86.0% to 82.0%. Feature B showed a comparable result. There was a continuous increase up to 74.5% (t_2_) in ICU 1, but a decrease from 78.0% (t_1_) to 72.5% (t_2_) in ICU 2. A detailed examination of the questionnaires revealed a heterogeneous understanding of the role of RNs. Primary nurses, for example, indicated a high level of responsibility for the nursing care process, while associated nurses often gave heterogeneous feedback in both ICUs, especially ICU 2.

### 4.2 Nursing Performance Indicators

**FIGURE 4:**
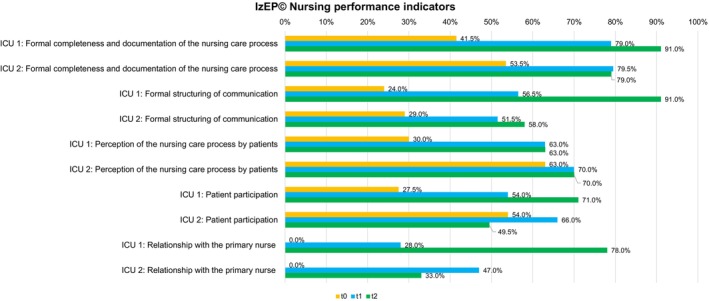
IzEP(c) nursing performance indicators for ICU 1 and ICU 2. ICU, Intensive care unit; IZEP(c), Instrument zur Erfassung von Pflegesystemen.

Nursing performance indicators showed a heterogeneous development in both ICUs (Figure 4). There was an increase in formal completeness and documentation of the nursing care process since t_0_ up to 91.0% (ICU 1) and 79.0% (ICU 2), but a small decrease in ICU 2 between t_1_ (79.5%) and t_2_ (79.0%). Formal structuring of communication increased in both ICUs over the three time periods, up to 91% (ICU 1) and 58% (ICU 2) in t_2_. Perception of the nursing care process by patients increased in t_1_, but stagnated in t_2_ in both ICUs (ICU 1: 63.0%; ICU 2: 70.0%). Patient participation and relationship with the primary nurse increased over all time periods in ICU 1 (71.0% and 78.0%), but decreased between in t_1_ (66.0% and 47.0%) and t_2_ (49.5% and 33.0%) in ICU 2.

## 5 Discussion

This quantitative study evaluated the process of PN implementation in two ICUs of a university hospital over a time period of 18 months by using IzEP(c). Overall, the results indicate positive developments in both ICUs after 6 months of PN implementation. After 12 months, PN was successfully implemented in ICU 1, whereas ICU 2 did not reach full implementation. In both ICUs, potential for improvement could be shown.

We followed the handbook of IzEP(c), met all requirements and included a large number of questionnaires with 192 participants. However, the involvement of external professional groups often varies between hospitals and units. Speech therapists, psychologists and neurologists are only present in the participating ICUs when their expertise is required.

Both ICUs practiced individual nursing in t_0_ with heterogeneous characteristics. ICU 1 finally reached an overall level of 83% and practiced PN. A development could also be shown in ICU 2, but despite reaching 69%, PN was not completely practiced. However, Fröhlich et al. [13] reported 75% in two ICUs and 68% in one ICU, while Krüger et al. [12] reached 83.5%. In this context the results can be considered acceptable, especially considering that PN is only practiced from the third day of patients' stay and not with admission on ICU. However, no relatives confirmed that a single nurse was responsible for the entire stay in t_2_. Maybe, as discussed by Fröhlich et al. [13], this was because they also met the associated nurses due to the different shifts and therefore could not confirm this statement. Nevertheless, from the perspective of the relatives, there was a positive development, but further efforts are needed. The primary nurse has an important role in the context of family‐centred care. Family members should be involved in bedside care [9] and first positive signs from the implementation of PN, compared with individual care, could be identified [15].

Around 40% of possible patients were included in PN in ICU 1, while only around 25% could be included in ICU 2. This circumstance may also have contributed to the overall result in ICU 2. In general, both ICUs need to work on admitting more patients in PN.

The detailed ICU profile of ICU 1 showed an increase in nearly all dimensions of feature A, but a small decrease of 5% in nursing care process. Minor deviations should not be overinterpreted, however, and should still be considered in the analysis. Fröhlich et al. [13] and Krüger et al. [12] also showed small isolated declines between t_1_ and t_2_, but in different features. Nursing managers play an important role in the development and implementation of PN, and primary nurses need their support [25]. Moreover, fostering open communication between leaders and staff is essential to enhancing nurse participation [26]. Within the context of the nursing care process, maybe the nursing management in ICU 1 made minor adjustments while they filled out their questionnaires in t_1_ and t_2_, as also discussed by Krüger et al. [12]. The same applies to ICU 2. Here, a small decrease in understanding of roles could be found between t_1_ and t_2_ in feature A. Feature B showed an overall increase between t_0_ and t_2_ in ICU 1.

Nevertheless, the distances to feature A vary, with this feature generally being ranked higher. Communication, in particular, was ranked one‐fifth lower by participants than by nursing managers. Recent studies have identified communication barriers in the context of PN, arising from differing attitudes and expectations between nurses and physicians [7, 8] or primary and associated nurses [8]. On the other hand, communication is a key element for professional patient [2, 27, 28] and relative care [9, 29] especially in PN [1]. In ICU 2, all features B decreased between t_1_ and t_2_. This may be due to a reduced presence of primary nurses in practice. In the context of implementation, PN leaders are also needed, who represent the project in daily practice [18]. Compared to ICU 1, in ICU 2 both primary nursing managers studied nursing management and nursing science in part‐time. For this reason, they were not permanently present on their ICU to support their staff day by day. This factor may also have exerted a negative influence on the study results.

Nursing performance indicators in ICU 1 also showed a good development over the three time periods. Maybe also the practiced nursing visits supported the implementation process [30] on ICU 1. Implementation of this intervention is still planned in ICU 2 for 2026. Integration of relatives in nursing visits is possible and should be discussed and taken into account in the context of family‐centred care [9]. Nevertheless, in ICU 2 a reduction of nursing performance indicators was observed only in patient participation and relationship with the primary nurse. This may also have been due to the partial absence of primary nurses and must be addressed at first by the nursing management. It underscores the importance of a well‐realised communication between primary nurses, patients and especially relatives.

### 5.1 Strengths and Limitations

A strength of our study is the integration of two large ICUs in a university hospital for implementation of PN. Furthermore, we used IzEP(c) as specific and validated questionnaires for data collection.

Our study also has limitations. We only considered quantitative results for this analysis. For an overall process evaluation additional qualitative results could be helpful, but an integration in this paper was not practicable. Moreover, IzEP(c) is only available in German and is practiced as interviews. This could have led to an interviewer bias. However, all nurses who conducted the data collection had previously received comprehensive training. Additionally, critically ill patients are not always able to understand complex questions. Patients with mechanical ventilation or sedation were not included in this study and their relatives could not be asked to participate.

### 5.2 Implications for Practice and Further Research

First of all, it will be a key role for especially the nursing management in ICUs to further support their nursing teams and also the interdisciplinary staff. Maybe the evaluation in our study needs a fourth or fifth time period in ICU 2. As an alternative to IzEP(c) in German‐speaking countries for a faster process, the validated questionnaire of Kien et al. [31] could be used for medical staff to evaluate the implementation process on PN. Internationally, further different tools, like Ward Organisational Feature Scales (WOFS) [32] or the Nursing Practice Model [33] are available in English [22]. A further analysis of qualitative results could help for a better understanding and to confirm or refute our results.

In general, as recommended by MRC Framework, implementing PN as a complex intervention should be evaluated in a mixed‐methods design with a process evaluation [17]. Furthermore, a deeper and better understanding of the attitude of or advantages for relatives through PN should be studied.

## 6 Conclusions

PN on ICU needs continuous support from nursing managers for successful implementation. Our results showed a progress from individual nursing to PN, although full implementation was not achieved in ICU 2. Nevertheless, ICU 2 also showed a change in nursing organisation and PN was practiced in included patients. However, the remaining challenge is ensuring that all those involved recognise and support this across the entire ICU.

## Funding

The study was financed by the own funds of the Heart and Diabetes Center NRW, Ruhr University Bochum.

## Ethics Statement

The ethics committee of the medical faculty of the Ruhr University Bochum, Germany, based in East Westphalia, has given a favourable ethics vote on November 18th 2022 (No 2022‐987).

## Consent

Before the beginning of the study, the participants signed an informed written consent form.

## Conflicts of Interest

The authors declare no conflicts of interest.

## Supporting information


**Table S1:** Standards for Quality Improvement Reporting Excellence Guideline (Squire 2.0).
**Table S3:** Included participants and questionnaires in IzEP(c).
**Figure S4:** IzEP(c) overall ICU profile of ICU 1 and ICU 2 between t_0_ and t_2_.
**Table S5:** Rating of the nursing care situation by relatives.
**Table S6:** Rating of the nursing care situation by nurses.
**Table S7:** Patients on ICU 1 and ICU 2 between February 2024 and March 2025.

**Table S2:** Task profile of primary nurses and associated nurses.

## Data Availability

Data are available by the first author under consideration of aspects of protection of data of the participants.
