# Supplementary material for: Primary Nursing in Intensive Care Units
Source: Nurs Crit Care. 2026 Jan 20;31(1):e70325. doi: 10.1111/nicc.70325 (PMC12818099; doi:10.1111/nicc.70325)
Supplement: Supplementary file 1 — Table S1: Standards for Quality Improvement Reporting Excellence Guideline (Squire 2.0). Table S3: Included participants and questionnaires in IzEP(c). Figure S4: IzEP(c) overall ICU profile of ICU 1 and ICU 2 between t0 and t2. Table S5: Rating of the nursing care situation by relatives. Table S6: Rating of the nursing care situation by nurses. Table S7: Patients on ICU 1 and ICU 2 between February 2024 and March 2025. [file NICC-31-0-s002.docx]

# Electronic Supplements

**Title**

Primary Nursing in Intensive Care Units

**Authors**

Lars Krüger, Thomas Mannebach, Francesco Squiccimarro, Laura-Carina Kurz, Christian Höke, Almut Pörner, Benjamin Sarx, Christian Siegling, Esther Mertins, Tobias Becker, René Schramm, Jan Gummert, Volker Rudolph, Gero Langer, Franziska Wefer

**Content**

Table S1: Completed Standards for Quality Improvement Reporting Excellence
 Guideline (Squire 2.0) Checklist

Table S2: Task profile of primary nurses and associated nurses
 (Please see separate Microsoft Excel Table S2)

Table S3: Included participants and questionnaires in IzEP©

Figure S4: IzEP© overall ICU profile of ICU 1 and ICU 2 between t_0_ and t_2_

Table S5: Rating of the nursing care situation by relatives

Table S6: Rating of the nursing care situation by nurses

Table S7: Patients on ICU 1 and ICU 2 between February 2024 and March 2025

**Table S1: Standards for Quality Improvement Reporting Excellence Guideline (Squire 2.0)**

Reference:

Ogrinc G, Davies L, Goodman D, Batalden P, Davidoff F, Stevens D. SQUIRE 2.0 (Standards for QUality Improvement Reporting Excellence): revised publication guidelines from a detailed consensus process. BMJ quality & safety. 2016;25(12):986-92.

| **Text Section and Item**  **Name** | **Section or Item Description** | **Page** |
| --- | --- | --- |
| **Title and Abstract** |  |  |
| **1. Title** | Indicate that the manuscript concerns an initiative to  improve healthcare (broadly defined to include the quality, safety, effectiveness, patient-centeredness, timeliness, cost,  efficiency, and equity of healthcare) | Cover page |
| **2. Abstract** | 1. Provide adequate information to aid in searching and   indexing   1. Summarize all key information from various sections of the text using the abstract format of the intended publication or a structured summary such as: background, local problem, methods, interventions, results, conclusions | 1-2 |
| **Introduction** | *Why did you start?* |  |
| **3. Problem Description** | Nature and significance of the local problem | 4-5 |
| **4. Available knowledge** | Summary of what is currently known about the problem, including relevant previous studies | 4-5 |
| **5. Rationale** | Informal or formal frameworks, models, concepts, and/or theories used to explain the problem, any reasons or  assumptions that were used to develop the intervention(s), and reasons why the intervention(s) was expected to work | 5 |
| **6. Specific aims** | Purpose of the project and of this report | 5 |
| **Methods** | *What did you do?* |  |
| **7. Context** | Contextual elements considered important at the outset of introducing the intervention(s) | 5-6 |
| **8. Intervention(s)** | 1. Description of the intervention(s) in sufficient detail that others could reproduce it 2. Specifics of the team involved in the work | 6-7 |
| **9. Study of the Intervention(s)** | 1. Approach chosen for assessing the impact of the intervention(s) 2. Approach used to establish whether the observed outcomes were due to the intervention(s) | 7-8 |
| **10. Measures** | 1. Measures chosen for studying processes and outcomes of the intervention(s), including rationale for choosing them, their operational definitions, and their validity and reliability 2. Description of the approach to the ongoing assessment of contextual elements that contributed to the success, failure, efficiency, and cost 3. Methods employed for assessing completeness and accuracy of data | 7-8 |
| **11. Analysis** | 1. Qualitative and quantitative methods used to draw inferences from the data 2. Methods for understanding variation within the data, including the effects of time as a variable | 7-9 |
| **12. Ethical**  **Considerations** | Ethical aspects of implementing and studying the intervention(s) and how they were addressed, including, but not limited to, formal ethics review and potential conflict(s)  of interest | 9 |

| **Results** | *What did you find?* |  |
| --- | --- | --- |
| **13. Results** | 1. Initial steps of the intervention(s) and their evolution over time (*e.g.*, time-line diagram, flow chart, or table), including modifications made to the intervention during the project 2. Details of the process measures and outcome 3. Contextual elements that interacted with the intervention(s) 4. Observed associations between outcomes, interventions, and relevant contextual elements 5. Unintended consequences such as unexpected benefits, problems, failures, or costs associated with the intervention(s). 6. Details about missing data | 9-11 |
| **Discussion** | *What does it mean?* |  |
| **14. Summary** | 1. Key findings, including relevance to the rationale and specific aims 2. Particular strengths of the project | 11 |
| **15. Interpretation** | 1. Nature of the association between the intervention(s) and the outcomes 2. Comparison of results with findings from other publications 3. Impact of the project on people and systems 4. Reasons for any differences between observed and anticipated outcomes, including the influence of context 5. Costs and strategic trade-offs, including opportunity costs | 11-13 |
| **16. Limitations** | 1. Limits to the generalizability of the work 2. Factors that might have limited internal validity such as confounding, bias, or imprecision in the design, methods, measurement, or analysis 3. Efforts made to minimize and adjust for limitations | 13 |
| **17. Conclusions** | 1. Usefulness of the work 2. Sustainability 3. Potential for spread to other contexts 4. Implications for practice and for further study in the field 5. Suggested next steps | 14 |
| **Other information** |  |  |
| **18. Funding** | Sources of funding that supported this work. Role, if any, of the funding organization in the design, implementation,  interpretation, and reporting | Cover page |

(The page numbers refer to the accepted manuscript version.)

**Table S3: Included participants and questionnaires in IzEP©**

| **Included participants and questionnaires in IzEP©** | | | | | | | |
| --- | --- | --- | --- | --- | --- | --- | --- |
|  | **ICU 1  (n t_0_)** | **ICU 2 (n t_0_)** | **ICU 1 (n t_1_)** | **ICU 2 (n t_1_)** | **ICU 1 (n t_2_)** | **ICU 2 (n t_2_)** | **Overall questionnaires (n)** |
| **Nurses** | 15 | 15 | 15 | 15 | 15 | 15 | 90 |
| **Therapeutic professions^1^** |  | | | | | | |
| Physiotherapist | 2 | 2 | 2 | 2 | 2 | 2 | 12 |
| Physician | 1 | 1 | 1 | 1 | 1 | 1 | 6 |
| **External professional groups** |  | | | | | | |
| Neurologist | 1 | 1 | 1 | 1 | 1 | 1 | 6 |
| Psychologist | 1 | 1 | 1 | 1 | 1 | 1 | 6 |
| Speech therapist | 1 | 1 | 1 | 1 | 1 | 1 | 6 |
| **Nursing management ICU** | 1 | 1 | 1 | 1 | 1 | 1 | 6 |
| **Care directorate** | 1 | 1 | 1 | 1 | 1 | 1 | 6 |
| **Patients** | 6 | 6 | 6 | 6 | 6 | 6 | 36 |
| **Relatives** | 3 | 3 | 3 | 3 | 3 | 3 | 18 |
| **Patient documentation** | 6 | 6 | 6 | 6 | 6 | 6 | 36 |
| **Duty roster of nurses** | 6 | 6 | 6 | 6 | 6 | 6 | 36 |
| **Overall participants** |  | | | | | | 192 |
| **Overall questionnairs** |  | | | | | | 264 |

ICU: Intensive care unit; IzEP©: Instrument zur Erfassung von Pflegesystemen
^1^In IzEP© physiotherapists receive the same questionnaire as physicians and both are named in the category *Therapeutic professions*.

**Figure S4: IzEP© overall ICU profile of ICU 1 and ICU 2 between t_0_ and t_2_**

<10%: no clear rule in nursing organization; 10–40%: functional nursing; 41–75%: individual nursing; > 75–100%: primary nursing

ICU: Intensive care unit; IzEP©: Instrument zur Erfassung von Pflegesystemen

**Table S5: Rating of the nursing care situation by relatives**

| **Which of the following statements best describes the situation of your relative?** | | | | | | |
| --- | --- | --- | --- | --- | --- | --- |
|  | **ICU 1 (n t_0_)** | **ICU 2 (n t_0_)** | **ICU 1 (n t_1_)** | **ICU 2 (n t_1_)** | **ICU 1 (n t_2_)** | **ICU 2 (n t_2_)** |
| I don't know, I can't judge | 1 |  |  | 2 |  |  |
| … that in almost every shift a different nurse is responsible for   her/him | 1 | 1 | 1 |  |  |  |
| … that several nurses are responsible for her/him in each shift |  |  |  |  |  |  |
| … that the same nurse is responsible for her/him across   multiple shifts (e.g., early shifts) | 1 | 2 | 1 | 1 | 3 | 3 |
| … that a single nurse is responsible for her/him for the entire   stay |  |  | 1 |  |  |  |

ICU: Intensive care unit

**Table S6: Rating of the nursing care situation by nurses^1^**

| **Which of the following statements best describes the situation on your ICU?** | | | | | | |
| --- | --- | --- | --- | --- | --- | --- |
|  | | |  |  |  |  |
|  | **ICU 1 (n t_0_)** | **ICU 2 (n t_0_)** | **ICU 1 (n t_1_)** | **ICU 2 (n t_1_)** | **ICU 1 (n t_2_)** | **ICU 2 (n t_2_)** |
| **On our ICU, patients mainly experience...** |  | | | | | |
| … that in almost every shift a different nurse is responsible for  her/him | 8 | 6 | 8 | 2 | 1 | 3 |
| … that several nurses are responsible for her/him in each shift |  |  |  |  |  | 1 |
| … that the same nurse is responsible for her/him across multiple  shifts (e.g., early shifts) | 7 | 9 | 4 | 12 | 10 | 9 |
| … that a single nurse is responsible for her/him for the entire stay |  |  | 3 | 1 | 4 | 2 |

ICU: Intensive care unit
^1^One example of different items in context of the practiced nursing organization model in IzEP© questionnaire for nurses

**Table S7: Patients on ICU 1 and ICU 2 between February 2024 and March 2025**

| **Patients on ICU 1 and ICU 2 between February 2024 and March 2025** | | |
| --- | --- | --- |
|  | | |
|  | **ICU 1 (n)** | **ICU 2 (n)** |
| Patients | 2015 | 1866 |
| ICU stay >3 days | 216 | 289 |
| Included patients in PN | 80 | 69 |
| Time of admission on ICU to inclusion in PN (days/IQR) | 8 (3 to 14) | 5 (2 to 9,5) |
| Duration of PN (days/IQR) | 17 (9 to 61) | 9 (6 to 19) |

ICU: Intensive care unit; IQR: Interquartile Range; PN: Primary Nursing
